# Urgent vs. early-start peritoneal dialysis: patients' profile and outcomes

**DOI:** 10.1590/2175-8239-JBN-2020-0011

**Published:** 2020-06-29

**Authors:** Viviane Calice-Silva, Bruna C. Tonial, Helen C. Ferreira, Fabiana B. Nerbass

**Affiliations:** 1 Fundação Pró-Rim, Joinville, SC, Brasil.; 2 Universidade da Região de Joinville - Univille, Escola de Medicina, Joinville, SC, Brasil.

**Keywords:** Peritoneal Dialysis, Kidney Failure, Chronic, Renal Replacement Therapy, Diálise Peritoneal, Falência Renal Crônica, Terapia de Substituição Renal

## Abstract

**Introduction:**

Peritoneal dialysis (PD) has been considered a safe option of therapy in end-stage renal disease patients with urgent need of dialysis. Recently, it was proposed that Urgent-Start-PD (US-PD) be defined when PD starts within 72 hours after catheter placement and “early start” PD (ES-PD) when PD starts between 3 and 14 days after. We aimed to compare demographic and clinical characteristics between patients in US-PD and ES-PD as well as 30-day complications, 6-month hospitalization, and dropout rate.

**Methods:**

Adult patients starting PD within 14 days after catheter insertion (October/2016 - February/2019) were included and divided into US-PD group and ES-PD group based on the their PD initiation time. Clinical and demographic data, fill volume for the first PD session, 30-day complications, 6-month hospitalization, and dropout rate were assessed.

**Results:**

In our study, 72 patients were analyzed (US-PD=40, ES-PD=32) with mean age of 53.2±15.2 years old. No differences between US-PD and ES-PD regarding demographic characteristics, 30-day complications, 6-month hospitalization, and dropout events were found. The most frequent short-term complication in patients who started PD urgently was leakage. The most common cause of dropout was transfer to HD.

**Conclusion:**

Fifty five percent of our sample started PD less than 72 hours after catheter insertion. The lack of difference in the measured outcomes compared to patients that had therapy initiated after this period encourages the use of urgent PD when needed.

## RESUMO

**Introdução:**

A diálise peritoneal (DP) tem sido considerada uma opção segura de terapia em pacientes com doença renal terminal com necessidade urgente de diálise. Recentemente, foi proposto que a DP de início urgente (US-PD) fosse definida quando a DP iniciar dentro de 72 horas após o implante do cateter, e a DP de "início precoce" (ES-PD) quando a DP iniciar entre 3 e 14 dias após o procedimento. Nosso objetivo foi comparar características demográficas e clínicas entre pacientes em US-PD e ES-PD, bem como complicações em 30 dias, internação por 6 meses e taxa de saída do tratamento.

**Métodos:**

Pacientes adultos iniciando DP em até 14 dias após a inserção do cateter (outubro/2016 - fevereiro/2019) foram incluídos e divididos no grupo US-PD ou ES-PD com base no tempo de início da DP. Dados clínicos e demográficos, volume de preenchimento na primeira sessão de DP, complicações em 30 dias, hospitalização por 6 meses e taxa de saída foram avaliados.

**Resultados:**

Em nosso estudo, 72 pacientes foram analisados (US-PD = 40, ES-PD = 32) com média de idade de 53,2 ± 15,2 anos. Não foram encontradas diferenças entre US-PD e ES-PD em relação às características demográficas, complicações em 30 dias, hospitalização por 6 meses e saída do tratamento. A complicação de curto prazo mais frequente nos pacientes que iniciaram a DP com urgência foi o extravasamento de líquido peritoneal. A causa mais comum de saída foi a transferência para HD.

**Conclusão:**

Mais da metade da nossa amostra iniciou a DP menos de 72 horas após a inserção do cateter. A falta de diferença nos desfechos mensurados em comparação com os pacientes que iniciaram o tratamento após esse período incentiva o uso de US-PD quando necessário.

## INTRODUCTION

Unplanned peritoneal dialysis (PD), also known as urgent-start PD (US-PD), has gained more attention in recent years due to its favorable short and long-term outcomes.^1^^-^ 3 At present, there is no consensus regarding US-PD definition.^4^ Most define it as therapy initiation within 14 days of PD catheter insertion, since the International Society for Peritoneal Dialysis (ISPD) and European Renal Best Practice (ERBP) guidelines suggested a break-in period after catheter placement of at least 15 days.^5^^,^ 6 This recommendation aims to minimize the risk of pericatheter or incisional leakages and allow patients training before starting PD at home.^7^ Recently, Blake and Jain proposed that the term “urgent-start” PD be reserved for patients with truly urgent clinical presentations requiring PD within 72 hours of catheter insertion. The more elective variant, where PD is started between 3 and 14 days after catheter insertion and may undergo hemodialysis (HD) previous to PD, is best termed “early-start PD”.^8^ Patients that would truly need urgent PD initiation would be a mix of those with unrecognized advanced chronic kidney disease (CKD) and those with recognized CKD, but with unexpected deterioration of the residual renal function. Ideally, those patients should start PD directly with no prior HD treatment. However, some authors suggest that in some situations PD could be contraindicated such as hyperkalemia with electrocardiogram alteration, hypervolemia and pulmonary edema with the need of mechanical ventilation and FiO_2_ ≥70%, and others. In that case, as mentioned above, if HD is necessary for compensation, PD starting after that would be considered as “early-start” and not urgent-start.^8^^,^ 9. In this single-institution retrospective study, we aimed to compare demographic and clinical characteristics of patients that started PD therapy defined as urgent- and early-start as well as 30-day complications and 6-month hospitalization and dropout rate.

## METHODS

### PATIENT SELECTION

Inclusion criteria comprised adult patients that started PD therapy up to 14 days after catheter insertion in our institution between October 2016 and February 2019, regardless of the need for hospitalization to start the therapy or not. Patients were placed on the urgent-start (US-PD) group if they had an urgent indication of renal replacement therapy (RRT) and started PD within 72-h after catheter insertion or early-start (ES-PD) group if PD initiated between 3 and 14 days. Patients that, for any reason, needed HD previously to PD start were also considered as early-start.

### PD TREATMENT

PD sessions, most of the time, started in the hospital, and right after clinical compensation the patient was discharged and maintained on intermittent PD (IPD) at the clinic. The number of days on IPD was individualized and varied from three to seven times a week. The dialysis was performed by the nurse team and patients and caregivers started the training during this period. Considering the fill volume, most patients received in the first PD session a 2,000 mL fill volume regardless of being in the urgent- or early-start groups. Only those with signs of leakage during the catheter implantation procedure received a lower fill volume (never less than 1,600 mL). If patients did not present complications during the first 2-3 sessions, the fill volume was increased progressively until achieving a volume considered satisfactory for clearance, according to the patients’ size and tolerance.

### CATHETER IMPLANTATION

In this study, the majority of catheters were placed by one of the two PD nephrologists by modified Seldinger technique. In few cases, a surgeon performed the procedure by mini-laparotomy, mini-laparotomy, and video-laparoscopy. The decision of who would perform the procedure was mainly based on staff availability and patients’ characteristics.

### DATA COLLECTION

Dialysis records were reviewed to obtain clinical and demographic data, fill volume prescribed for the first PD session, 30-day complications (leakage, bleeding, catheter tip migration, and peritonitis), 6-month hospitalization events, and dropout rate. Peritonitis was confirmed with PD fluid cell count and positive peritoneal fluid cultures. Leakage was recorded if any amount of PD fluid was drained through the catheter exit site. Bleeding was recorded if present in the PD catheter exit site or with presence of hemoperitoneum after catheter implantation, and catheter tip migration was identified by abdominal x-ray to investigate fluid drainage problems. The mean days of IPD was also collected for both groups.

### OUTCOMES

Outcomes assessed were first 30-day complications, 6-month hospitalization events, and 6-month dropout.

## RESULTS AND DISCUSSION

**Figure 1:**
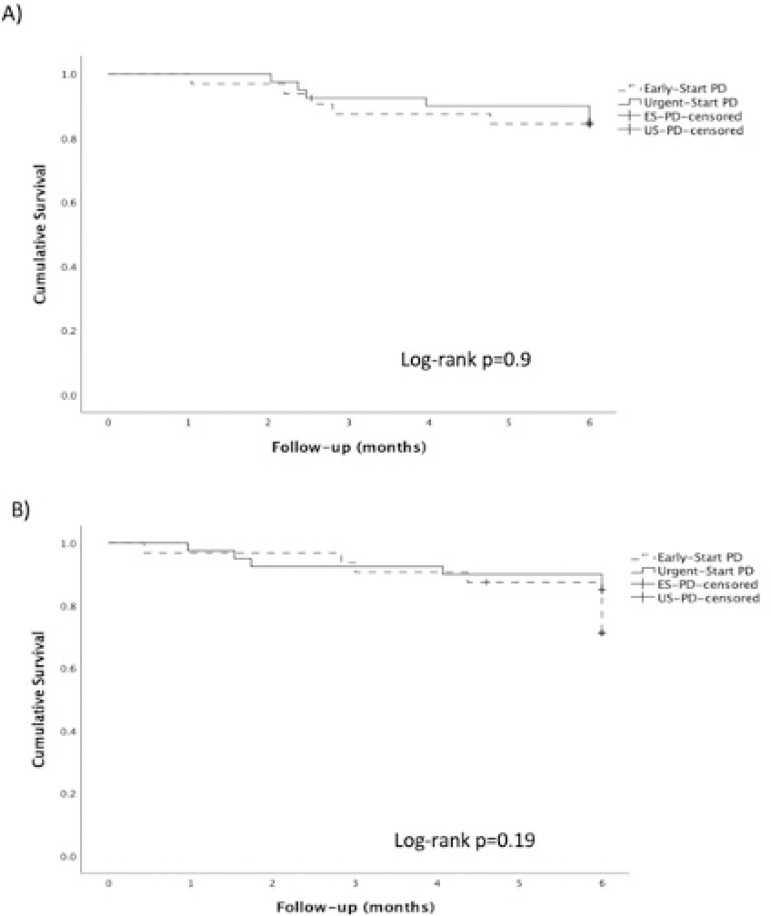
Kaplan-Meier curves for dropout (A) and hospitalization (B), comparison between urgent-start PD vs early-start PD in the first 6 months of therapy.

In this retrospective analysis, we did not find differences between urgent-start and early-start PD regarding demographic characteristics, 30-day complications, and 6-month hospitalization and dropout events (Table 1 and Figure 1).

**Table 1.** Comparison of characteristics and outcomes between urgent-start and early-start PD groups.

	Total (n=72)	Early-start PD (n=32)	Urgent-start PD (n=40)	P
Age (years)	53.2 ± 15.2	53.8 ± 16	52.8 ± 14.6	0.6
Male, n (%)	36 (50)	17 (53.1)	19 (47.5)	0.53
Skin color white, n (%)	66 (92)	29(90.6)	37 (92.5)	0.55
More than 8 years at school, n (%)	32 (45)	17 (53.1)	22(55)	0.53
Hypertension, n (%)	65 (90)	29 (90.6)	36 (90)	0.62
DM, n (%)	30 (42)	13 (40.6)	17 (42.5)	0.53
Previous HD, n (%)	17 (24)	17 (53.1))	0	<0.001
Technique (Seldinger), n (%)	47 (65)	18 (56.3)	29 (72.5)	0.12
First treatment fill volume (mL)	1882 ± 133	1872 ± 130	1890 ± 137.4	0.57
PD initiation after catheter implantation (days)	2 (0-9)	4 (0-9)	1(0-3)	<0.001
30-day complications, n (%)	15 (21)	6(18.8)	9 (22.5)	0.46
Leakage	8 (11)	5 (15.6)	3 (7.5)	0.24
Bleeding	2 (3)	1 (3.1)	1 (2.5)	0.87
Catheter tip migration	4 (6)	0 (0)	4 (10)	0.9
Peritonitis	1 (1)	0 (0)	1(2.5)	0.57
6-month hospitalization, n (%)	15 (21)	9(28.1)	6 (15)	0.14
6-month dropout, n (%)	11 (15)	5 (15.6)	6 (15)	0.6
Kidney transplant	1 (9.1)	1 (20)	0 (0)	
Transfer to HD	5 (45.4)	2 (40)	3 (50)	
Transfer to another center	1 (9.1)	0 (0)	1 (16.7)	
Death	4 (36.4)	2 (40)	2 (33.3)	

PD: peitoneal dialysis; DM: diabetes mellitus; HD: hemodialysis.

The most frequent short-term complication in patients who started PD urgently was catheter tip migration and leakage, as previously described by other studies.^10^^-^ 12 The most common cause of patient dropout was transfer to HD for both groups (two patients in ES-PD and three in US-PD). The second most common cause of dropout was death with no difference between groups, as previously shown in investigations that compared US-PD with planned-start PD (after 15 days of catheter insertion).^13^ It is important to mention that main reasons for hospitalizations during the first six months of treatment were cardiovascular causes, not related to PD complications (four patients in ES-PD and one patient in US-PD group). Only one patient in each group was hospitalized due to peritonitis during the follow-up period. In our service, all patients with peritonitis diagnosis started their treatment hospitalized.

According to this new classification, patients who underwent HD before PD were classified as an ES-PD, regardless of days between catheter placement and PD initiation. This was necessary mainly due to PD facility logistic factors, for example, if a patient needed dialysis urgently and nephrologists were not able to perform the catheter implantation or when there was a contraindication for PD at the moment of clinical evaluation.

The implementation of unplanned PD program is an excellent strategy to increase PD penetration, not only from the point of view of expanding the PD program but also from the perspective of optimizing the utilization of this RRT modality globally. As consistently demonstrated in many publications about the use of PD in acute kidney injury and nowadays in urgent ESRD patients, PD is a safe option of RRT start even in life-threatening conditions with secure and satisfactory results when performed by a dedicated and well-prepared team.^14^^-^ 17

In our institution, after almost three years, the number of patients increased 2.2 fold and prevalence of patients on PD compared to HD almost doubled (from 15 to 27%). In Brazil in 2017, only 6.9% of prevalent patients on chronic dialysis were on PD.^18^ Considering the lack of available sites for HD around the country, PD is a safe treatment to overcome this deficiency.

## CONCLUSION

Fifty five percent of our sample started PD with an urgent indication and within 72 hours after catheter insertion. The lack of difference in measured outcomes compared to patients that had therapy initiated after this period encourages the use of PD when urgent dialysis is needed.
