# Erratum

**DOI:** 10.1590/2175-8239-jbn-2020-0011er

**Published:** 2021-05-12

In the article “Urgent vs. early-start peritoneal dialysis: patients' profile and outcomes”, with DOI code number https://doi.org/10.1590/2175-8239-jbn-2020-0011, published at Brazilian Journal of Nephrology, ahead of print, 2020:

Where it was written: 

Viviane Calice-Silva^1,2^

http://orcid.org/0000-0002-9696-0529

Bruna C. Tonial^2^

http://orcid.org/0000-0002-7570-7241

Helen C. Ferreira^1^

http://orcid.org/0000-0003-0268-9617

Fabiana B. Nerbass^1^

http://orcid.org/0000-0001-9936-0185

^1^Fundação Pró-Rim, Joinville, SC, Brasil.

^2^Universidade da Região de Joinville - Univille, Escola de Medicina, Joinville, SC, Brasil.

Should read: 

Viviane Calice-Silva^1,2^

http://orcid.org/0000-0002-9696-0529

Bruna C. Tonial^2^

http://orcid.org/0000-0002-7570-7241

Pedro Eugênio Deboni Daudt^2^

https://orcid.org/0000-00029676-8254

Izabel Ribeiro^2^

https://orcid.org/0000-0003-3522-7232

Helen C. Ferreira^1^

http://orcid.org/0000-0003-0268-9617

Fabiana B. Nerbass^1^

http://orcid.org/0000-0001-9936-0185

^1^Fundação Pró-Rim, Joinville, SC, Brasil.

^2^Universidade da Região de Joinville - Univille, Escola de Medicina, Joinville, SC, Brasil.

